# PDCD-DAT – a global database of pyroclastic density current deposit field data

**DOI:** 10.1186/s13617-026-00167-6

**Published:** 2026-05-11

**Authors:** Joshua Brown, Rebecca Williams, Sarah Ogburn, Brittany Brand, Eric C. P. Breard, Sylvain Charbonnier, Natasha Dowey, Josef Dufek, Mark Jellinek, Ulrich Kueppers, Gert Lube, Pete Rowley

**Affiliations:** 1 School of Environmental and Life Sciences, University of Hull, Hull, UK; 2 School of Natural Sciences, Trinity College Dublin, Dublin, Ireland; 3 U.S. Geological Survey, Vancouver, WA USA; 4 Department of Geosciences, Boise State University, Boise, USA; 5 School of Geosciences, University of Edinburgh, Edinburgh, UK; 6 Department of Earth Sciences, University of Oregon, Eugene, USA; 7 School of Geosciences, University of South Florida, Tampa, USA; 8 Geography, Environment and Planning, Institute of Law and Social Sciences, Sheffield Hallam University, Sheffield, UK; 9 Department of Earth, Ocean and Atmospheric Sciences, University of British Columbia, Vancouver, Canada; 10 Department for Earth and Environmental Studies, Ludwig-Maximilians-Universität (LMU), Munich, Germany; 11 School of Agriculture and Environment, Massey University, Palmerston North, New Zealand; 12 School of Earth Sciences, University of Bristol, Bristol, UK

**Keywords:** Pyroclastic density current, Deposit, Database, Grain size data, Sedimentary structures, Volcanic hazard, Volcano

## Abstract

Pyroclastic density currents represent one of the deadliest hazards posed by active volcanoes. Analysis of their deposits provides valuable insights into their internal dynamics and informs numerical simulations of pyroclastic density currents which underpin many volcanic hazard assessments. We present PDCD-DAT, a global database of pyroclastic density current deposit characteristics compiled from peer-reviewed literature. The database includes both quantitative datasets (e.g., grain size, density, bedform dimensions, thickness) and qualitative descriptors (e.g., sedimentary structures, lithofacies). PDCD-DAT includes data from 85 source publications, covering 97 eruptions or eruptive phases, and 214 individual depositional units from 55 globally distributed volcanoes. Eruptions recorded in the database range from VEI 1–8. We highlight examples of potential applications of the database, which include (i) comparison of single deposit case studies to global datasets, (ii) informing input parameters and conditions for numerical and analogue models of pyroclastic density currents, (iii) validation of numerical and analogue models against a wide variety of natural case study deposit architectures, and (iv) estimating hazard impact metrics of pyroclastic density currents from past eruptions. We show that the database represents a useful tool for improving our ability to model pyroclastic density currents, predict their associated hazards, and understand the relationships between the internal dynamics of pyroclastic density currents and the properties of their deposits. PDCD-DAT is integrated with the FlowDat Mass Flow Database, which provides a sustainable platform for the database. We aim for PDCD-DAT to be expanded in the future through addition of pyroclastic density current deposit datasets from new field studies conducted by the volcanology research community.

**Supplementary Information:**

The online version contains supplementary material available at 10.1186/s13617-026-00167-6.

## Introduction

Pyroclastic density currents (PDCs) are mixtures of ash, gas, and rocks that form during explosive volcanic eruptions, via lava dome collapse, or following rainfall or gravitational failure of perched loose material. PDCs pose one of the greatest volcanic hazards to populations near active volcanic centres and are directly responsible for over 90,000 deaths since 1600 AD (Auker et al. 2013). The internal dynamics of PDCs cannot be directly observed and much of our understanding of their complex physics is inferred from analysis of the deposits they leave behind. For example, the sedimentary structures within PDC deposits (e.g., cross-stratification, grading) and changes in maximum clast/grain size and deposit thickness with distance from source are used to infer properties of the parent current, e.g., high or low particle concentration, turbulent or granular flow (e.g., Branney and Kokelaar 2002; Sulpizio et al. 2007; Brand et al. 2016; Palladino and Giordano 2019; Giordano et al. 2024). The interpretation of PDC dynamics from their deposits is important for reconstructing eruption processes at individual volcanoes and informing hazard assessments. Complementary insights into PDC internal dynamics can be obtained from numerical models and analogue experiments, which simulate PDCs at varying scales and degrees of complexity. However, our ability to use numerical and analogue models to test relationships between deposit properties and the currents and/or processes that formed them is limited by a lack of compiled quantitative datasets for different PDC deposit architectures to inform and validate against. Here, we address this limitation and present a first global compilation of PDC deposit characteristics.

Data obtained from PDC deposits, such as grain size distributions (GSDs) and particle shape and density, are key input parameters in numerical simulations of PDCs (e.g., Gueugneau et al. 2020; Esposti Ongaro et al. 2020; Calabrò et al. 2022), which are becoming increasingly important for hazard assessment at active volcanoes (e.g., Charbonnier et al. 2020; Esposti Ongaro et al. 2020; Gueugneau et al. 2024; Aravena et al. 2024). For example, the product of particle sphericity (a shape parameter) and Sauter mean diameter (calculated from the grain size distribution) can be used to estimate the permeability of complex volcanic mixtures, which controls the formation and diffusion of elevated gas pressure in multiphase models (Breard et al. 2019). Particle density influences particle settling velocity and therefore represents an important parameter for modelling sedimentation from PDCs (e.g., Dellino et al. 2008; Kelfoun 2017; Jones et al. 2023).

Deposit data can also be used to inform input parameters in benchmarking studies that compare the outputs of different PDC models against a solution (field-based, analytical, etc.) and assess their strengths and weaknesses. Ogburn and Calder (2017) used multiple physical and empirical models to simulate PDCs from Soufrière Hills Volcano (Montserrat, Lesser Antilles) and compared how well the different models reproduced characteristics of the natural PDC deposits (a field-based solution) such as runout and inundated area. Properties of the natural deposits, such as H/L (ratio of height descended (H) to PDC runout (L), volume, and planimetric area, were used to calculate model input parameters.

Field observations from natural PDC deposits (e.g., thickness, grain size, and deposit temperature during emplacement) can be compared with model outputs to validate the extent to which numerical and analogue models are able to realistically simulate natural PDCs (Charbonnier and Gertisser 2012; Charbonnier et al. 2013; Lube et al. 2015; Kelfoun et al. 2017; Brosch and Lube 2020). For example, Smith et al. (2020) show that bedforms produced in their analogue experiments simulating dense, granular flows have similar morphology and stoss side angles to bedforms in the Pozzolane Rosse ignimbrite deposits of Colli Albani volcano. Kelfoun et al. (2017) showed that their numerical simulations of PDCs were able to quite accurately reproduce key characteristics of PDC deposits from the 2010 eruption of Merapi volcano, including thickness and volume.

Studies of PDC deposits can also improve our ability to forecast the impacts associated with PDCs. The hazard potential of PDCs can be evaluated based on parameters such as the dynamic pressure, which is used to estimate whether PDCs will damage buildings (Zuccaro et al. 2008), and particle volumetric concentration, which influences whether humans caught in a PDC can survive (Baxter et al. 2017; Dellino et al. 2021a). Recent models allow these hazard impact metrics to be estimated for dilute PDCs from past eruptions based on deposit properties such as grain size, bedform dimensions, and particle shape (Dioguardi and Mele 2018; Dellino et al. 2021b). The estimated values for hazard impact metrics are assumed to be representative of the PDC at the location of the sampled outcrop (Dioguardi and Mele 2018; Dellino et al. 2021b) and represent time averages.

However, our ability to compare and collate comprehensive field datasets for integration with numerical, analogue, and hazard models is currently limited, because i) many studies of PDC deposits focus on a single eruption or sometimes an individual depositional unit; (ii) there is no standardised approach for documenting deposit characteristics; and (iii) there is no publicly available database of PDC deposit characteristics. Rather, many studies that attempt to use field data to inform or compare with either numerical or analogue models rely on single case studies (e.g., Charbonnier and Gertisser 2012; Salvatici et al. 2016; Kelfoun et al. 2017; Smith et al. 2020).

Several studies have collated data on deposit characteristics as a means for classification, or as an attempt to link deposit characteristics to flow processes (e.g., Walker 1971; 1983; Giordano and Cas 2021), but restricted the studied deposits to ignimbrites. The term ignimbrite has had a complex history since its first use by Marshall (1932) for rhyolitic sheets in New Zealand. Originally synonymous with welded tuffs, it has variably been defined or used to describe non-welded to rheomorphic tuffs, large volume deposits, pumice-rich tuffs, pumice- and ash-rich tuffs, and more recently has also included scoria- and ash-rich tuffs (e.g., Marshall 1932; Smith 1960; Walker 1983; Branney and Kokelaar 2002; Giordano and Cas 2021 and references therein). The term is typically understood to exclude deposits formed from the gravitational collapse of lava domes (i.e., BAFs/Block-and-ash-flows), and lateral blasts. We use the term ‘PDC deposit’ to describe any deposit formed from a pyroclastic density current, including any currents (regardless of composition, concentration, or mode of formation) that in the literature may be referred to as dense or dilute PDCs, pyroclastic flows, pyroclastic surges, or block-and-ash-flows, amongst other descriptors.

In this contribution, we present a global database of PDC deposit characteristics (PDCD-DAT), incorporating quantitative data (e.g., grain size, density, bedform dimensions) and qualitative descriptions of deposit appearance (e.g., sedimentary structures, lithofacies). PDCD-DAT is integrated with the FlowDat Volcanic Mass Flow Database (Ogburn 2012, 2025). Integration with FlowDat provides a long-term sustainable platform for our database, which will allow for it to be expanded in the future and enables users to explore relationships between PDC deposit characteristics and the bulk PDC characteristics (e.g., volume, area) and mobility metrics (e.g., runout length, H/L) already recorded in FlowDat.

PDCD-DAT can be used for a variety of applications, including (i) comparison of single deposit case studies to global datasets; (ii) informing numerical and analogue model input parameters; (iii) validation of numerical and analogue models against a wide variety of natural deposits; (iv) estimating hazard impact metrics of PDCs from past eruptions to inform hazard assessments; (v) identifying discrepancies between data required by modellers and the data most commonly collected and reported in PDC field studies. Therefore, the database represents a tool for improving our understanding of the dynamics of PDCs and our ability to model their complex physics and predict their associated hazards. We hope to expand and improve PDCD-DAT in the future through incorporation of additional existing studies and members of the volcanological research community submitting newly collected quantitative datasets.

## Construction and content

To determine the deposit properties to be recorded in the database, we compiled an initial list of quantitative measurements (e.g., grain size, componentry, bedform dimensions) and qualitative descriptors (e.g., sedimentary structures, lithofacies types) commonly used in the literature to document PDC deposits. Further properties were added to the database throughout the compilation process, to capture the wide variety of data types reported in field studies of PDC deposits.

The FlowDat database (Ogburn 2012; 2025) was used as a starting point to identify target PDC deposits and sources of PDC deposit data. We focused on eruptions in FlowDat for which quantitative data were already recorded for one or more of the following bulk PDC properties or mobility metrics: total flow bulk volume, total flow planimetric area, runout, and H/L. This approach was chosen to enable users of the database to explore the relationships between PDC deposit properties and other PDC properties/mobility metrics. We searched the literature for studies on PDC deposits associated with these eruptions and relevant peer-reviewed studies containing at least one form of quantitative data were incorporated into the database. FlowDat also contains data on eruption VEI, magma composition, and geographic location. Care was taken to ensure that deposit data for a global distribution of volcanoes and eruptions spanning a wide range of magnitudes and magma compositions were incorporated into the database.

We sourced additional data from the authorship teams’ publications, as well as studies known to the authorship team containing high-quality quantitative datasets and example datasets for the least frequently reported properties, e.g., grain shape, Sauter mean diameter. Incorporating such example datasets ensures that additional datasets reporting these properties can be easily added into the database in the future, without modifying the data import template. Data from publications suggested by the wider PDC research community were also incorporated, following discussions at the 2nd National Science Foundation community workshop ‘Benchmarking of PDC models and other avenues’ held in August 2024. PDCD-DAT does not include datasets from submarine or welded PDC deposits, which are often described using different metrics to non-welded subaerial deposits (Quane and Russell 2005).

The database currently includes data from 85 source publications, covering 97 eruptions or discrete phases within long-lived eruptions, and 214 individual depositional units. Seventy-two of the eruptions/eruption phases recorded in PDCD-DAT have associated data on PDC mobility metrics, VEI, and magma composition in FlowDat. Eruptions recorded in the database vary in magnitude from VEI 1–8 and have magma bulk compositions ranging from basaltic andesite to rhyolite, and trachybasalt to trachyte and phonolite. The database includes deposits from 55 volcanoes distributed across 6 continents (Additional File 4).

We acknowledge that PDCD-DAT does not represent a complete record of published PDC deposit data and that our database compilation strategy introduces some limitations to its potential use. For example, PDC deposits have been more extensively studied for volcanoes in some regions (e.g., Europe, North America) than others (e.g., Indonesia), hence PDCD-DAT contains data from multiple references for some volcanoes (e.g., Vesuvius), but none for other volcanoes that are known to produce frequent PDCs (but which are documented in FlowDat, e.g., Sinabung, Semeru). The lack of PDC deposit studies on volcanoes located in tropical/highly vegetated regions such as Indonesia may be due to poor exposure and high potential for erosion of PDC deposits in these regions (e.g., Carn, 1999), or less available funding and resources to conduct research compared with regions such as Europe.

Some PDC deposit properties (e.g., grain shape parameters) have far fewer entries than other properties that are more commonly reported (e.g., grain size). Although we have included at least one example dataset for all deposit properties recorded in the database, it is possible that our compilation strategy has overlooked some studies reporting the less-well-represented properties.

Eruptions of VEI 3–6 make up ~ 80% of eruptions/eruption phases recorded in the database; PDCs from very low and high magnitude eruptions are less-well-represented. This is likely a reflection of the lower preservation potential of smaller volume deposits associated with lower VEI eruptions (e.g., Cowlyn et al. 2020), or simply that small, frequent PDCs are less often studied, and the less-frequent occurrence of very high magnitude eruptions. At ocean island volcanoes, even high magnitude eruptions may be poorly preserved due to deposition into the surrounding ocean (e.g., Porreca et al. 2018). We hope that potential future expansion of the database will address some of these limitations, especially the low number of entries for certain deposit properties such as grain shape.

In PDCD-DAT, quantitative data is usually reported for individual samples/sampling locations or lithofacies, while qualitative observations may be reported for specific sampling locations or more generally for depositional units, sub-divisions of units, or lithofacies. While PDCD-DAT is integrated into the relational SQL FlowDat database (and will eventually be searchable on the web at https://flowdat.org/pdcd-dat/), we also provide a flat-file, spreadsheet version of the data (Additional File 1). This flat-file is organised into nine categories – Metadata, Grain size, Grain Characteristics, Componentry, Sedimentary Structures, Bedforms, Thickness, GSD Deconvolution, and Temperature (see also Additional File 6). Each category is represented by a table containing a series of columns which record relevant PDC deposit properties. Each row in the tables represents an individual data entry (e.g., grain size measurement for a given sample, sedimentary structure(s) within a unit, bedform dimensions at a given distance from the vent). The PDC deposit properties recorded in each category are briefly described below – the full list of properties and their definitions are provided in Additional File 2.

*1)* The *Metadata* table records details of source publications and metadata associated with the data recorded in other tables, including the volcano and eruption date, vent and sampling location co-ordinates (using global co-ordinate systems, either latitude-longitude or UTM), the “PDC type” (e.g., concentrated, surge) as determined by the study authors, and the names and unique IDs of depositional units, sampling locations, sample names/identifiers and lithofacies (See Additional File 2 for database column definitions and explanation of the ID system used to associate data across tables and with FlowDat).

*2)* The *Grain Size* table records grain size distributions (GSDs) (in phi units), the most frequently reported statistical parameters used to describe grain size (median diameter, sorting coefficient, F1 weight percentages) and maximum juvenile (pumice, scoria, or dense juvenile blocks in BAF deposits) and lithic clast sizes (see Additional File 2 for definitions). The methods/equations used by source publication authors to calculate statistical parameters and determine maximum clast sizes, and the grain size range analysed for each sample, are reported to allow users to assess comparability of different datasets. Users are encouraged to refer to source publications for further details of the methods used to measure grain size. Raw GSD data are reported in 1 phi intervals to maintain consistency. If source publications reported data in 0.5 phi intervals, the data have been grouped into 1 phi bins accordingly. The notes column records additional relevant details, including whether more precise 0.5 phi interval data are available in the source publication, and grain sizes which were observed/measured but excluded from reported GSDs by authors, e.g., “blocks > -6 phi”.

3) The *GSD Deconvolution* table contains example datasets for less frequently reported measures of grain size. These include statistical parameters (median diameter, sorting coefficient) for individual components (juveniles, lithics) and statistics for grain sub-populations obtained via deconvolution of polymodal grain size distributions.

4) The *Grain Characteristics* table records measurements of individual grain/clast characteristics including (bulk) density and shape parameters (e.g., aspect ratio, Fourier Shape Analysis morphological coefficients) and the equations used to calculate these values.

*5)* The components of PDC deposit samples are often specific to a particular volcano (e.g., ripped up fragments of the substrate lithology). To facilitate comparison between deposits from different locations, the *Componentry* table records the proportions of the general component categories “juveniles,” “lithics,” and “crystals”. For the vast majority of samples documented in the database, components were explicitly assigned to one of these three categories in the source publication. We note that the definitions of “juvenile” and “lithic” may vary between studies and the reported values simply reflect the interpretation of the source publication authors. The *Componentry* table also records the grain size range analysed for componentry, in phi units, and whether the values were reported as wt% or proportions, to allow users to assess comparability of datasets.

6) The *Sedimentary Structures* table contains columns titled with general terms used to describe structures in PDC deposits, e.g., massive, inverse grading, cross-stratification. In many source publications, sedimentary structures were described for depositional units as a whole, rather than at individual sampling locations. In this scenario, all sample data entries from a given unit were assigned the relevant sedimentary structure. The “Notes” column is used to provide additional details of sedimentary structure distribution within a depositional unit, e.g., “lower half of unit inverse graded, upper half normally graded”. Quantitative data associated with sedimentary structures, such as thicknesses of strata and angles of cross-stratification are also recorded.

7) The *Bedforms* table records quantitative measurements of bedform dimensions and features, e.g., length/wavelength, stoss and lee angle, plus relevant contextual information such as the underlying depositional slope angle, where reported. In Additional File 2, we provide typical definitions for bedform measurements. For example, “wavelength” is typically used to refer to the distance between the crests or troughs of two adjacent/periodic bedforms, whereas length is typically used to refer to the distance between the base of the stoss and lee sides of a single bedform. We recommend that users check the definitions for bedform measurements used in source publications (if provided by their authors), which may differ slightly from the typical definitions listed in Additional File 2, when comparing bedform datasets.

8) The *Thickness* table records depositional unit thicknesses as either measured values or a range. Contextual information, including whether the top and base of a unit was exposed and the underlying topography at locations where thickness was measured (e.g., valley-confined vs. overbank), is also recorded where available.

*9)* The *Temperature* table records estimated emplacement temperatures for PDC deposits and the methods used to calculate them.

### Strategy for reporting of data

All events (eruptions or phases within long-lived eruptions), depositional units, sub-units, and lithofacies are assigned unique IDs in the database. Where a data entry refers to a sample analysis (e.g., grain size measurement, componentry), the associated sample name/code from the source publication is listed in the “sample name/identifier” column (present in all tables of the database, Additional File 1) and given a unique ID. If a sample name was not provided in the source publication (e.g., for data obtained from a table reporting only maximum clast size vs. distance), we assigned a sample name in the “sample name/identifier” column (e.g., ML1 for a measurement of maximum lithic size at a given distance) to provide an identifier for each data entry. We also assigned a name in the “sample name/identifier” column to measurements associated with a unique location, which were not directly associated with a sample (e.g., measurements of unit thickness or bedform dimensions at a given distance from the vent). All unique samples/sampling locations in the database were also assigned a unique sample ID number.

Where these metadata (event, unit, subunit, lithofacies, sample, sampling location, or a subset of those) form a unique combination, we have implemented unique “Data Unit IDs” to make it easy to trace directly associated data entries (e.g., grain size measurements and componentry from the same sample) across all data sheets. In some cases, data such as thicknesses or bedform measurements were reported for a unit or eruption in the source publication but were not explicitly associated with individual samples (measured for grain size, etc.) or sampling locations. Therefore, some data entries do not have unique “Data Unit IDs” but can still be connected with associated datasets from the same unit/eruption in other sheets using the other metadata (e.g., unit, subunit). The names of depositional units, sub-units, and sample/lithofacies names in the database exactly match the source publications wherever possible, to allow users to further investigate specific datasets or samples with ease.

The “PDC type” column reflects the terminology used by source publication authors to describe the type of current which formed the deposit (e.g., “block-and-ash flow”, “surge”, etc.). The terminology used in the database to record sedimentary structures matches the majority of source publications, though the exact choice of wording describing the same feature may differ between sources (e.g., “parallel” vs. “planar” stratification).

Quantitative data (e.g., distances, thicknesses, grain size, componentry, etc.) were recorded directly from published tables of values or the source publication text, wherever possible. For deposit thickness, only numerical values were recorded in the database – measurements listed in the format “centimetre-decimetre”, without explicit values, were excluded. For source publications where quantitative data were only presented in figures, we manually extracted the data using the free online tool WebPlotDigitizer (https://apps.automeris.io/wpd4/, (Rohatgi 2015). The “data source” column(s) in each table denote whether the data was obtained from a table, figure, text, or “estimated from figure” if extracted using WebPlotDigitizer.

The most common type of data extracted from figures were GSDs. Manual calibration of plot axes and manual selection of data points, combined with the low resolution of some figures, led to small inaccuracies in extracted GSDs (totals above or below 100%). Therefore, all GSDs extracted from figures were normalised so that the sum of all grain size fractions totalled 100%. We assessed the reproducibility of GSD extractions from figures using WebPlotDigitizer by performing 10 GSD extractions on the same figure, containing a GSD composed of 9 grain size fractions (-5 to 4 phi). The totals (prior to normalisation) of extracted GSDs ranged from 100.2 to 101.8%. The relative standard deviation (expressed as (2SD/mean) * 100) of the normalised values for each grain size fraction was < 4%, which confirms the reproducibility of our method for GSD extraction. When compiling the database, we found that uncertainties on quantitative data from PDC deposits are rarely reported, hence we do not list them for most of the properties recorded in the database.

## Utility and discussion

### Range of data recorded in PDCD-DAT

**Fig. 1:**
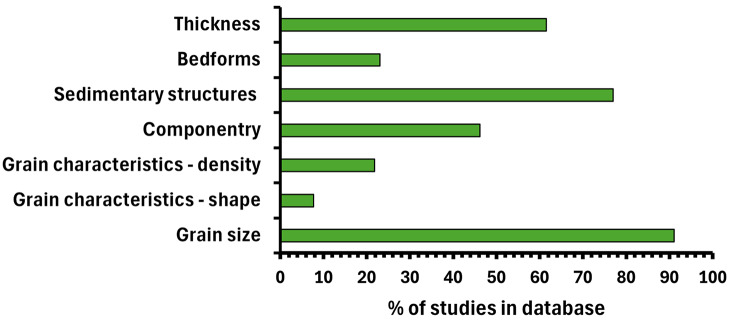
Percentage of studies in PDCD-DAT, excluding those included solely to record deposit emplacement temperatures, from which data corresponding to each category were extracted (*n* = 79). A further six studies were included in the Temperature category – no other data were obtained from these six studies

Figure 1 shows the percentage of studies in the database, excluding those included solely to record deposit emplacement temperatures, from which data corresponding to each category were extracted. Grain size represents the most frequently recorded data category (91% of studies), followed by sedimentary structures (77%). Density (22%) and grain shape parameters (8%) are the least frequently recorded data categories (see also Additional File 5).

**Fig. 2:**
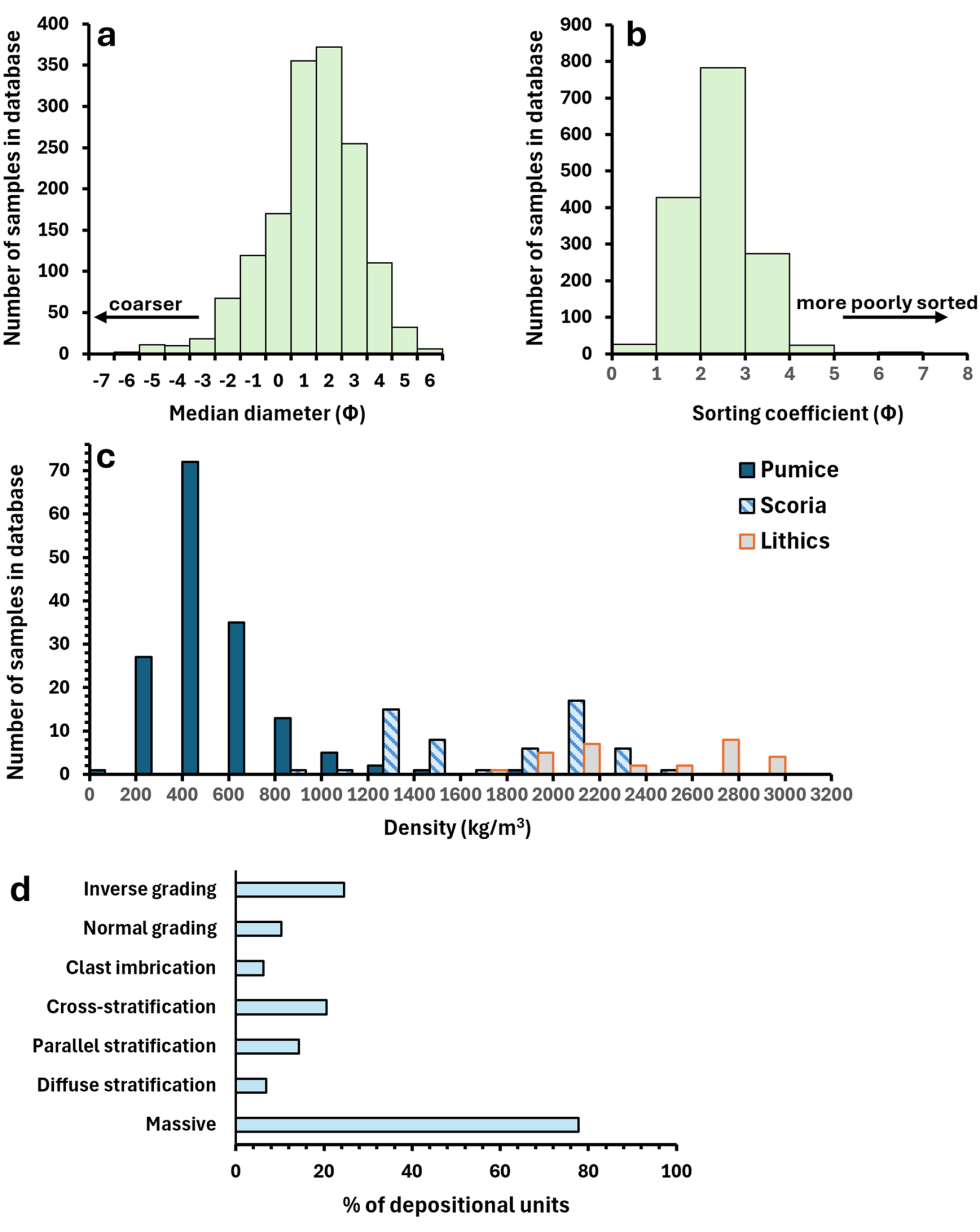
Distribution of (**a**) median diameter, (**b**) sorting coefficient and (**c**) clast density values for all individual pyroclastic density current (PDC) deposit samples recorded in PDCD-DAT. Clast density values are taken from the “avg” columns (Additional File 1) for pumice, scoria, and lithic density. (**d**) Percentage of depositional units recorded in PDCD-DAT displaying each type of sedimentary structure. Note that some units are associated with more than one sedimentary structure

**Fig. 3:**
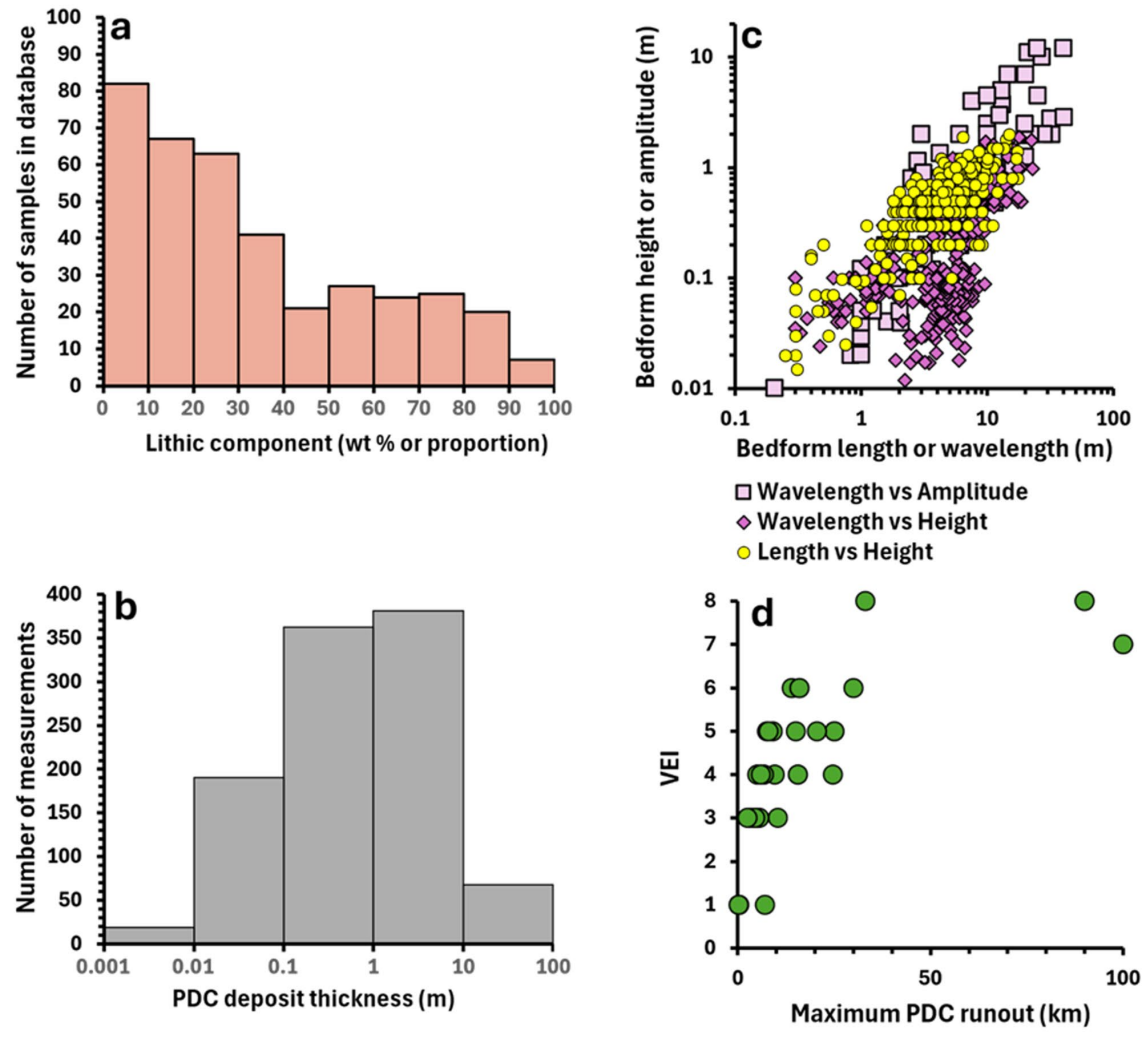
(**a**) Distribution of percentage lithic component values for all individual pyroclastic density current (PDC) deposit samples recorded in PDCD-DAT. Note that percentage lithics is reported as wt% or proportion (based on clast/grain counts) depending on the source publication (Additional File 1). (**b**) Distribution of PDC deposit thickness measurements (m) recorded in PDCD-DAT. (**c**) Distribution of bedform measurements recorded in PDCD-DAT. Data are grouped by the different measurement combinations used to document bedforms – wavelength vs. amplitude, wavelength vs. height, length vs. height. (**d**) Volcanic Explosivity Index (VEI) vs. maximum runout values from the FlowDat database (Ogburn 2012; 2025) for all eruptions in PDCD-DAT which have associated data for these two metrics in FlowDat

Figures 2 and 3 illustrate the range and distribution of data recorded in PDCD-DAT. Individual samples of PDC deposits cover a wide grain size range, with median diameters between − 7 and 6 phi and sorting coefficients between < 1–7 (Fig. 2, a, b). It should be noted that there are significant differences in the grain size ranges measured to obtain GSDs between studies (Additional File 1), which influences the distribution of median diameter and sorting coefficient values derived from these GSDs in the database. The majority of recorded PDC deposits (~ 78% of depositional units) are either partly or entirely massive (Fig. 2, d). Inverse grading is the most frequently reported sedimentary structure (recorded for 25% of depositional units), followed by cross-stratification (21%) (Fig. 2, d). The diverse componentry of PDC deposits is illustrated by the proportion of reported lithics, which varies from 0 to 99% (Fig. 3, a), though > 50% of PDC deposit samples contain < 30% lithics. PDC deposit thicknesses recorded in the database span four orders of magnitude, ranging from < 1 cm to 80 m (Fig. 3, b). Bedform wavelengths and amplitudes vary from 0.2–40 m and 0.01–12 m respectively (Fig. 3,c), whereas bedform lengths and heights span a slightly narrower range of 0.25–17.5 m and 0.01-2 m.

To give some examples of PDC deposits at the extreme ends of these data ranges: Charbonnier and Gertisser (2011) report the largest median diameter of -7.12 phi for unit L1A of the 14th June 2006 eruption of Merapi; Saucedo et al. (2010) report unit F3 with 99% lithics of the 1913 eruption of Colima; and Silleni et al. (2024) report a thickness of 80 m for the 39 ka Campanian Ignimbrite, Campi Flegrei. Estimated PDC deposit emplacement temperatures recorded in the database range from 170 to 480 °C.

### Applications of the database

In this section, we highlight some of the potential applications of PDCD-DAT using examples.

#### Informing, validating and benchmarking numerical and analogue models of PDCs

The database provides a valuable resource for reducing uncertainties associated with the choice of input parameters in some numerical and analogue models of PDCs. Grain size/GSDs, grain density and shape represent key input parameters in many numerical simulations of PDCs (e.g., Kelfoun et al. 2017; Gueugneau et al. 2020; Esposti Ongaro et al. 2020; Calabrò et al. 2022) and analogue experiments (e.g., Lube et al. 2015; Breard and Lube 2017; Smith et al. 2020). For example, Kelfoun et al. (2017) showed that their model outputs for total area covered by deposits and runout distance increased by ~ 20% and 13% respectively for a 50% decrease in particle diameter used in the model simulation. However, it is important to take into consideration that porous volcanic clasts change shape and size during PDC transport following clast-clast or clast-substrate interaction (Figueiredo et al. 2025). The database can be used to select input parameters for individual eruptions to inform models aiming to reproduce PDCs formed during past events.

Comparison of modelling results with PDC deposit data provides a useful tool for validating numerical and analogue models of PDCs. Determining the extent to which models reproduce the features of natural PDC deposits may demonstrate how accurately these models simulate natural PDCs and depositional processes. Datasets such as thickness and grain size variations with distance from source are key deposit characteristics that can be compared with model outputs to evaluate the performance of numerical models (Charbonnier and Gertisser 2012; Kelfoun et al. 2017; Gueugneau et al. 2020; Tadini et al. 2021). The integration of PDCD-DAT with FlowDat enables users to obtain additional data commonly used for numerical model validation, such as runout length and planimetric area (Widiwijayanti et al. 2009; Kelfoun et al. 2017; Gueugneau et al. 2020) and link these to the data for grain size, thickness, etc. PDCD-DAT enables numerical models to be calibrated against PDC deposits displaying a wide range of characteristics, from eruptions of different magnitudes (Fig. 3d). Therefore, PDCD-DAT can support the modelling community to determine the most reliable models for assessing the hazards posed by PDCs with specific characteristics and/or during different magnitude eruptions.

Similarly, comparison of the sedimentary structures, grain size distributions, and bedform appearance and dimensions in deposits formed in analogue experiments with natural PDC deposits can be used to confirm whether experiments can successfully replicate the transport and depositional processes of natural PDCs (Dellino et al. 2007; Lube et al. 2015; Brosch and Lube 2020; Smith et al. 2020). PDCD-DAT will enable analogue modellers to validate experiments which produce any of the common sedimentary structures/lithofacies reported in PDC deposits. Analogue experiments have the potential to provide new insights into the relationships between sedimentary structures/lithofacies/bedforms and the properties and internal processes of the currents which formed them, ultimately improving our ability to interpret natural PDC deposits.

Datasets for specific eruptions could also be used to select input parameters for benchmarking exercises, which compare the outputs of different numerical models against a defined solution e.g., a natural deposit (e.g., Ogburn and Calder 2017). Such benchmarking exercises can be used to evaluate the strengths and weaknesses of different models (e.g., how accurately do the models simulate natural PDC inundation, computational cost/time) to determine the scenarios in which they are best applied, e.g., as part of a procedure for rapid syn-eruption forecast of PDC inundation in a crisis.

#### Linking qualitative and quantitative PDC deposit data

Lithofacies and sedimentary structures exhibited by deposits (e.g., massive lapilli-tuff, cross-stratified tuff) are often used to infer conditions at the substrate-parent current interface, for example the common association of cross-stratified deposits with dilute and/or turbulent currents (e.g., Branney and Kokelaar 2002; Giordano et al. 2024). Our database offers an opportunity to interrogate whether these qualitative descriptors can be associated with certain values or ranges in quantitative deposit properties, such as grain size, for deposits from a large number of eruptions.

**Fig. 4:**
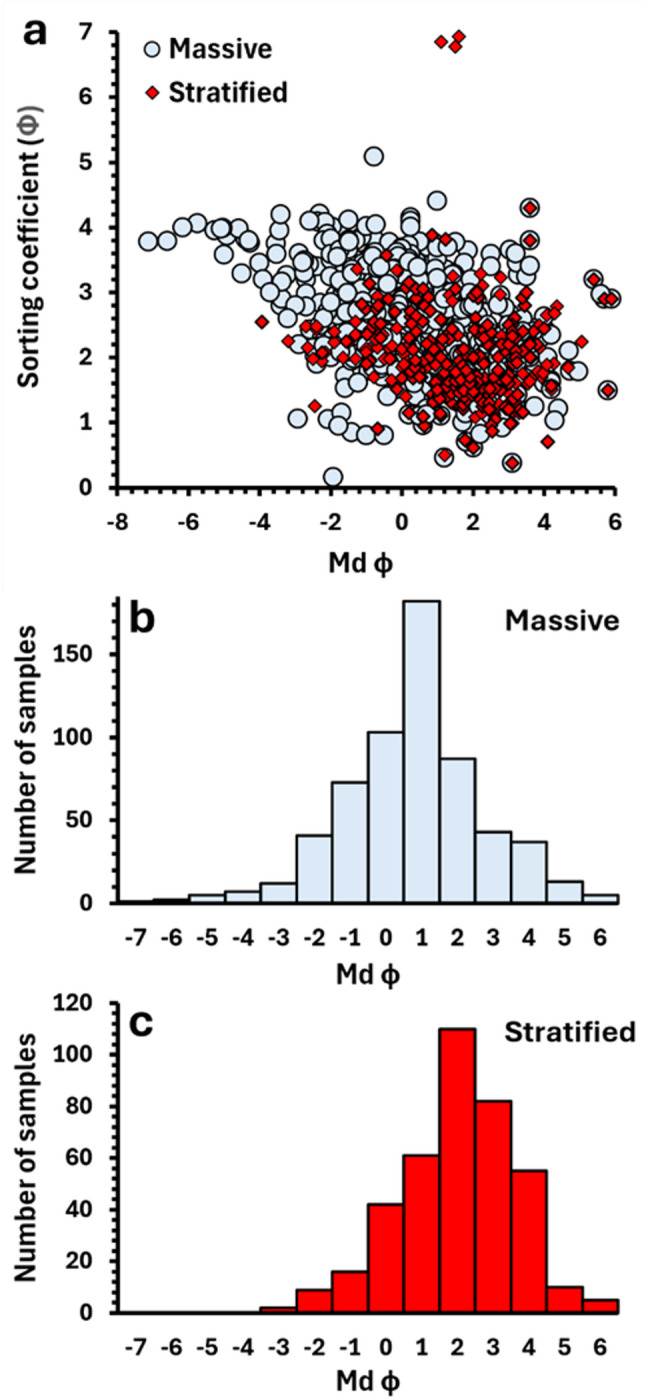
**(a)** Median diameter (Md φ) vs. sorting coefficient (phi units) for all PDC deposit samples in the database associated with massive (*n* = 611 individual samples), and parallel or cross-stratified (*n* = 392) sedimentary structures. **(b)** Distribution of massive deposit median diameters. **(c)** Distribution of stratified deposit median diameters. Note that the median diameter and sorting coefficient values shown were calculated by source publication authors using multiple different equations and/or software (e.g., Inman 1952; Folk and Ward 1957; GRADISTAT (Blott and Pye 2001). The corresponding grain size distributions are composed of varying grain size ranges and may have been measured in full or half phi units

A complete analysis of the database contents is beyond the objectives of this publication, but as an example of this application, we compare the grain size statistical parameters (median diameter and sorting coefficient) of PDC deposits displaying massive vs. parallel/planar stratified and cross-stratified sedimentary structures in Fig. 4a.

All data points reflect the values reported by source publication authors (as opposed to being calculated in this study). The reported median grain size and sorting of massive and stratified deposits show significant overlap, though massive deposits extend to larger median grain sizes and show a greater range in sorting compared with most stratified deposits (Fig. 4). We note that not all data plotted are directly comparable due to the different equations employed by source publication authors to calculate statistical parameters and differences in the grain size ranges measured to obtain the corresponding GSDs (see the “grain size” tab, Additional File 1). Despite this limitation, the general trend shown could provide a guideline for the most representative median grain size(s) (or grain size distributions) to use in analogue experiments aiming to simulate PDCs which form either massive or stratified deposits.

Source publication authors often infer that a PDC deposit was formed by a parent current with specific characteristics (recorded in the “PDC type” column of the Metadata tab, Additional File 1). The database captures a varied terminology that has evolved as our understanding of PDCs evolves (for a recent review, see Lube et al. 2020). Broadly, currents described in publications in the database are categorised according to end-member definitions referring to the particle concentration of the current, that can be grouped as “concentrated” (including “granular”) or “dilute” (including “surge”). The term “block-and-ash flow (BAF)” is used for concentrated currents that formed deposits composed of mostly juvenile blocks in an ash matrix, predominantly from gravitational collapse from a lava dome (Brown and Andrews 2015; Giordano et al. 2024). The term “lateral blast” (or blast surge) is commonly used in studies of the 1980 Mount St. Helens eruption (e.g., Fisher 1990) to describe a PDC formed during a laterally directed explosion. The term “pyroclastic flow” is also commonly used, and it is unclear whether this is a general term or should be interpreted to mean concentrated current (e.g., different to “surge”). Some studies simply use the term “PDC” and do not use further terminology implying the characteristics of the parent current.

**Fig. 5:**
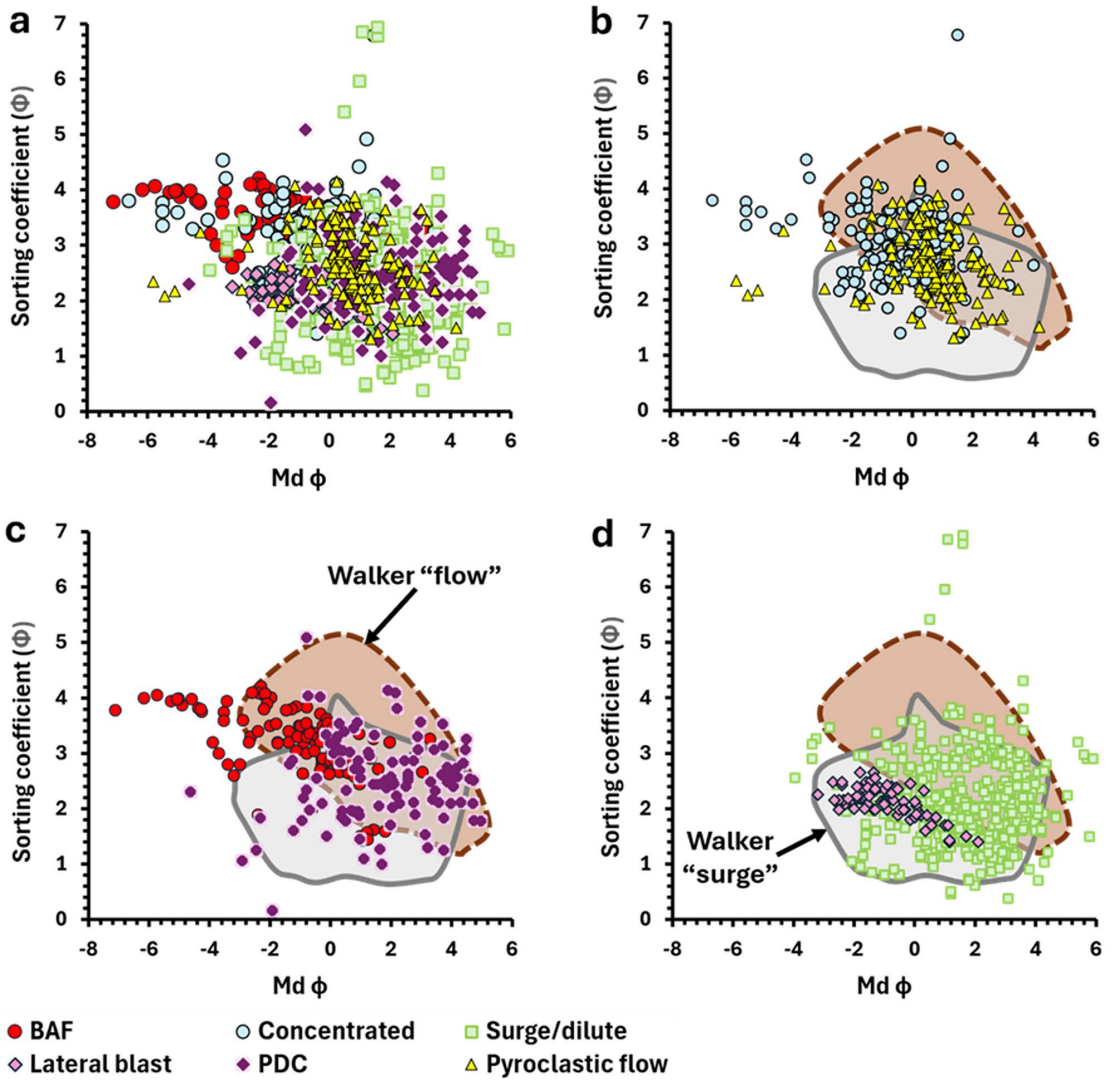
**a**) All data for median diameter (Md φ) vs. sorting coefficient (phi units) in PDCD-DAT for deposits associated with a named “type” of pyroclastic current - “block-and-ash-flow (BAF)” (n = 121 individual samples), “surge”/“dilute current” (n = 440), “concentrated current” (n = 180), “lateral blast” (n = 73), “pyroclastic flow” (n = 269), “pyroclastic density current (PDC)” (n = 112). **b**), **c**), **d**) The PDCD-DAT data for different named “types” of pyroclastic current are compared with the “pyroclastic surge” and “pyroclastic flow” fields of Walker (1983). Note that the median diameter and sorting coefficient values shown were calculated by source publication authors using multiple different equations and/or software (e.g., Inman 1952; Folk and Ward 1957; GRADISTAT (Blott and Pye 2001). The corresponding grain size distributions are composed of varying grain size ranges and may have been measured in full or half phi units

We show median diameter vs. sorting coefficient for PDC deposits inferred to have formed from different types of parent current in Fig. 5, following the plots of Walker (1971, 1983) initially used to distinguish “pyroclastic flow” and “pyroclastic surge” deposits. The PDCD-DAT datasets for “pyroclastic flow” and “surge/dilute” extend beyond the equivalent fields defined by Walker (1971, 1983) and show a greater degree of overlap in grain size and sorting compared with the Walker (1971, 1983) “flow” and “surge” fields. Overall, Fig. 5 demonstrates that the qualitative terminology used by source publication authors which implies parent current characteristics may not be reflected by clear differences in the reported quantitative properties of their deposits such as sorting and grain size. We note that these grain size statistical parameters may not accurately represent polymodal GSDs, such as those of many BAF deposits, and are not always directly comparable due to differences in the methods used to obtain GSDs and calculate statistical parameters (Additional File 1).

There has been increasing recognition that a single depositional unit may vary in lithofacies both laterally and vertically, leading to a tendency to group different lithofacies together into one unit, with a PDC type designated to the unit as a whole. Increasingly, a range of deposit parameters are used to determine whether a current is dilute (akin to surge) or concentrated (akin to pyroclastic flow). Altogether this would logically lead to a much larger range of grain size and sorting in these domains. Therefore, despite their common use, “Walker”-type plots may not represent a reliable tool for comparing PDC deposits or inferring parent current characteristics. Future work could use the database in combination with modelling to investigate this further by comparing grain size and sorting statistics for different volcano types, eruption volumes, or current/deposit parameters such as H/L and density.

#### Comparing individual volcanoes and eruptions

PDCD-DAT offers a powerful tool for comparing PDC deposits based on a wide range of criteria, to identify similarities and differences between deposits from different volcanoes, eruptions, or “types” of parent current. Subsequent investigations into the factors controlling common trends in PDC deposit characteristics, and unique trends observed at individual volcanoes, have potential to yield improved understanding of fundamental aspects of PDC behaviour.

**Fig. 6:**
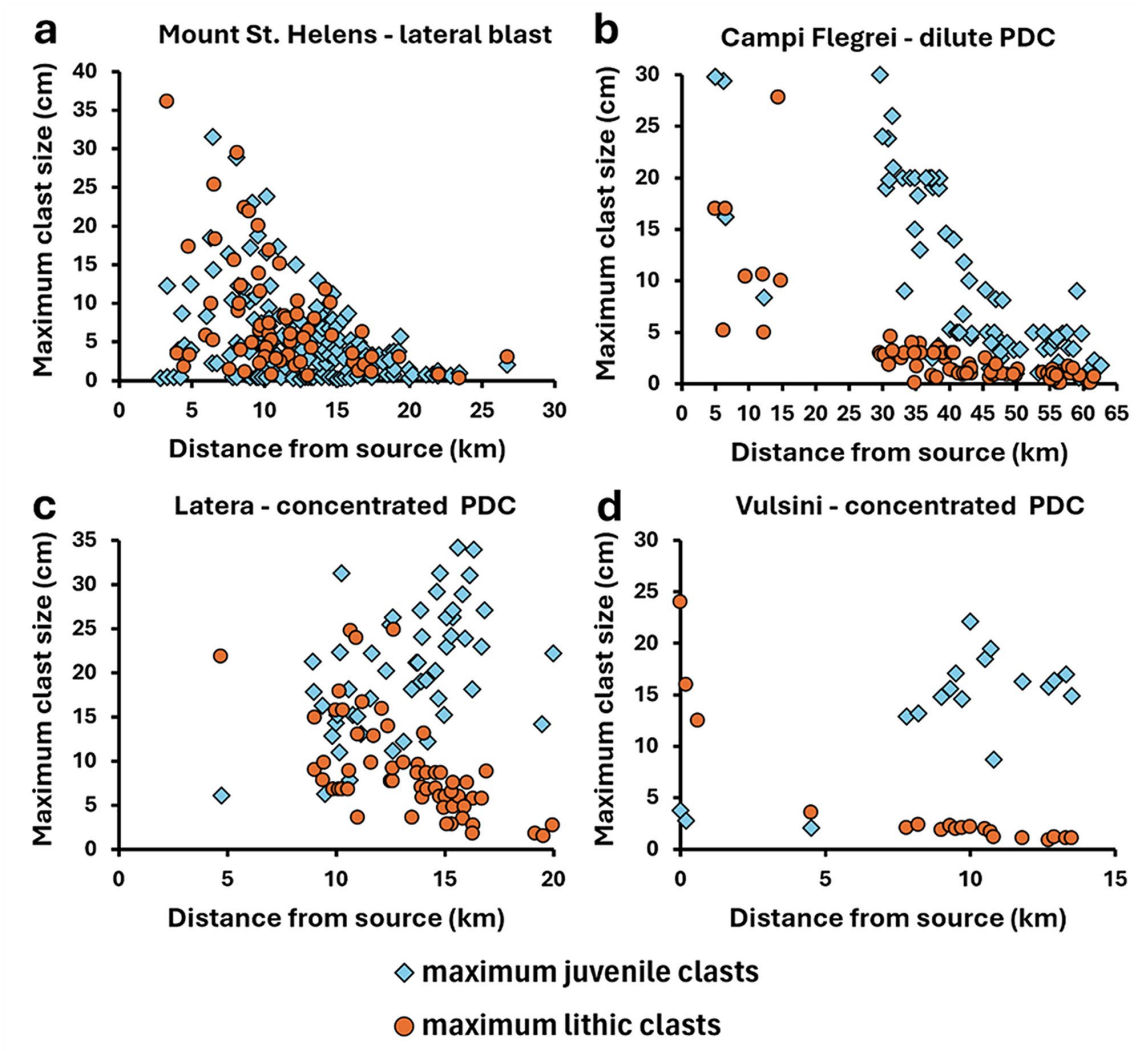
Maximum juvenile and lithic clast size vs. distance from source/vent for deposits from four eruptions. **(a)** Mount St. Helens - data from the 18th May 1980 eruption (Fisher 1990). **(b)** Campi Flegrei – data from the 39 ka Campanian Ignimbrite eruption (Silleni et al. 2024). **(c)** Latera – data from the Arlena di Castro flow unit, erupted from the Latera Volcanic Complex, Vulsini, at 0.23 Ma (Palladino and Giordano 2019). **(d)** Vulsini – data from the Orvieto-Bagnoregio ignimbrite, erupted from Vulsini at 333 ka (Palladino and Pettini 2020)

As an example of this application, in Fig. 6 we compare trends of maximum juvenile and lithic clast size vs. distance from source/vent for deposits from four eruptions. Maximum clast sizes decrease with distance from source in deposits inferred to have formed from dilute currents (Mount St. Helens, Campi Flegrei, Fig. 6a, b). Deposits from two eruptions from the Vulsini volcanic district, Italy, show contrasting trends of decreasing lithic and increasing juvenile clast size with distance (Fig. 6c, d), which are interpreted to reflect deposition from concentrated currents (Palladino and Giordano 2019; Palladino and Pettini 2020). Future experimental and modelling studies could aim to reproduce these trends for dilute and concentrated currents to quantify the physical characteristics of the parent currents that produce them (e.g., flow density) and associated ranges in hazard impact metrics (e.g., dynamic pressure) (Palladino and Giordano 2019).

**Fig. 7:**
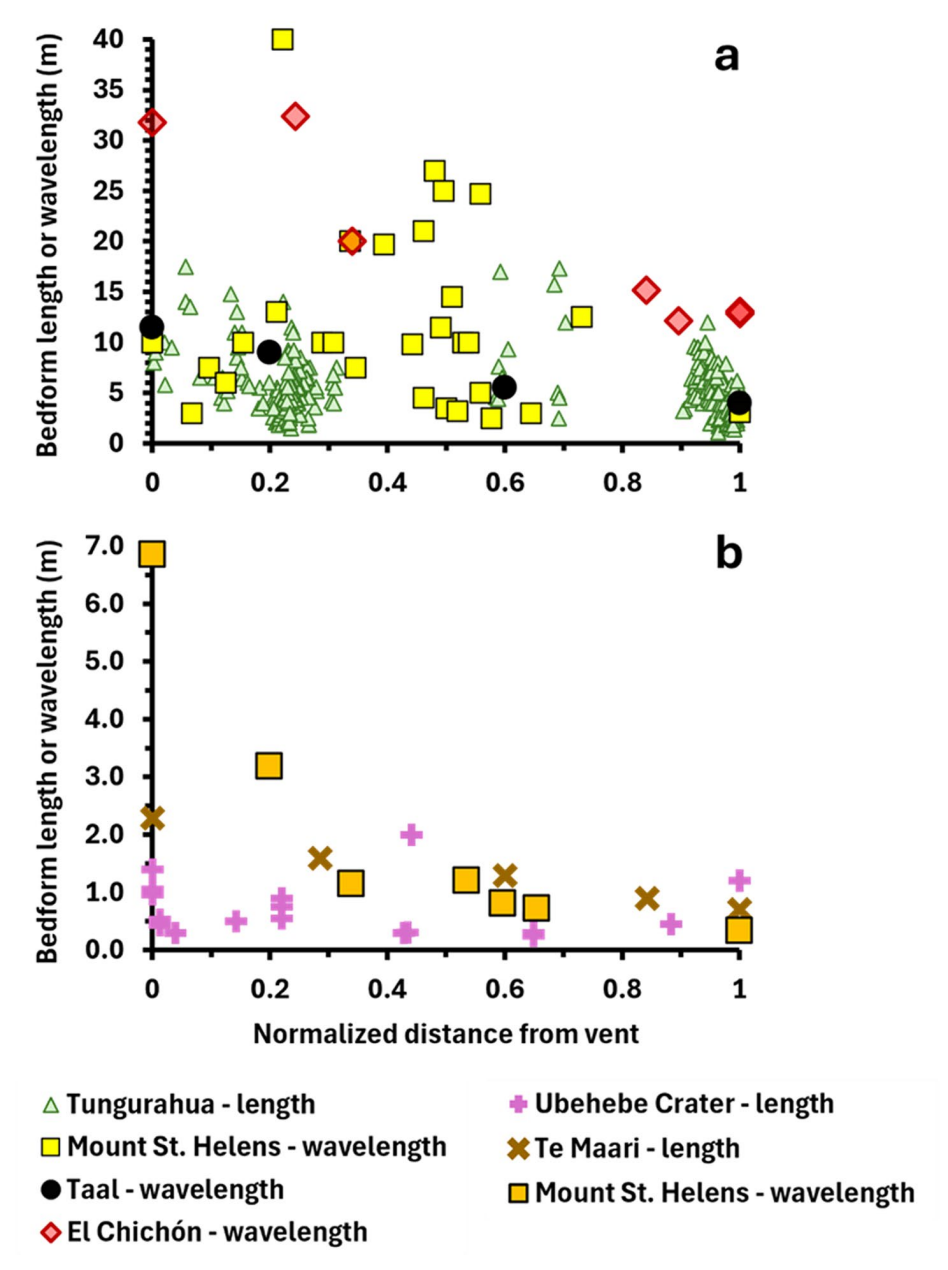
**a**), **b**) Trends of bedform length or wavelength vs. distance from vent for PDC deposits from six different volcanoes. The distances over which bedform measurements were taken vary significantly between volcanoes. To facilitate comparison, we normalised the bedform distances from vent for each volcano, based on the most proximal and most distal locations at which bedforms were measured. Normalised distances were calculated using the following equation: “Normalised distance from vent = (bedform measurement location distance – most proximal bedform measurement location distance)/(most distal bedform measurement location distance-most proximal bedform measurement location distance). Wavelength refers to the distance between crests or troughs of periodic bedforms in a single stratigraphic unit. Length refers to the distance between the base of stoss and lee side of a single bedform. Tungurahua – data from the August 2006 eruption (Douillet et al. 2013). Mount St. Helens, USA – data from the 18th May 1980 eruption (Druitt 1992; Brand et al. 2016). Taal, Phillipines – data from the 1965 eruption (Waters and Fisher 1971). El Chichón, Mexico – data from the 4th April 1982 eruption (Sigurdsson et al. 1987). Upper Te Maari Crater, Tongariro, New Zealand – data from the 6th August 2012 eruption (Breard et al. 2015). Ubehebe Crater, USA – data from deposits formed ~ 2.1 ka (Valentine et al. 2022)

Figure 7 shows that the length and wavelength of bedforms in PDC deposits from Taal, El Chichón, Mount St. Helens, Te Maari (Tongariro) and Ubehebe Crater volcanoes generally decreases with distance from vent. The controls on this trend are poorly constrained, though it has been suggested that particle concentration, current velocity, and current thickness may play a role (Brand et al. 2016). There is some evidence that bedform morphologies and scales can be quantifiably related to current characteristics (e.g., Dellino et al. 2020, 2021b; Smith et al. 2020). The presence of this trend in the deposits of five different volcanoes implies that the factors controlling bedform length/wavelength may reflect processes common to many PDCs and is an important avenue for future exploration.

#### Estimating the hazard potential of past PDCs

The hazard potential of PDCs can be estimated by calculating hazard impact metrics, such as average dynamic pressure during the passage of the current at a given location (i.e., particle volume concentration and velocity) and the flow temperature near the substrate. These metrics can be estimated for past eruptions from deposit characteristics recorded in the database, including bedform wavelength and median diameter (grain size), for example using the equations provided by Dellino et al. (2021b). More advanced models, such as “PYFLOW_ 2.0”, can be used to calculate hazard impact metrics from stratified deposits inferred to have formed from dilute currents, using thickness, grain size, density, and shape input data (Mele et al. 2015; Dioguardi and Mele 2018). The database can be used to obtain these parameters for some stratified deposits and identify other stratified deposits for which additional data could be obtained to facilitate these calculations. Metrics such as flow front velocity can be inferred from the size (e.g., maximum juvenile or lithic) and density of blocks reported from PDC deposits (Roche 2015; Roche et al. 2016).

#### Identifying gaps in data collection required for PDC modelling

Although we acknowledge that PDCD-DAT does not reflect a complete record of existing literature on PDC deposits, nor a random sample of deposits, it can be used to provide a guide for reporting of and comparison between different types of PDC deposit data.

Grain size, density, and shape represent the main input parameters for numerical models of PDCs which can be derived directly from deposits (e.g., Dellino et al. 2008; Kelfoun et al. 2017; Esposti Ongaro et al. 2020; Calabrò et al. 2022). The low frequency of density and particularly shape measurements in PDCD-DAT (Fig. 1, Additional File 1, Additional File 5) suggests that it may not always be possible for modellers to obtain accurate constraints on these parameters from existing field data. Particle shape data, for example, sphericity and circularity, are required for calculation of the drag coefficient parameter used in many numerical models of PDCs (e.g., Dellino et al. 2008; Kelfoun et al. 2017; Dioguardi and Mele 2018; Gueugneau et al. 2020), which may otherwise be estimated by a trial and error approach which explores a range of possible values (Kelfoun et al. 2017; Gueugneau et al. 2020). Therefore, more frequent reporting of particle shape datasets in future field studies of PDC deposits will increase the amount of accurate data available to modellers.

#### Limitations of existing PDC datasets

PDCD-DAT can be used for a variety of applications, but users face some limitations due to the lack of standardised approaches to reporting PDC deposit (meta) data. For example, GSDs are determined using a variety of methods (e.g., dry and wet sieving, laser diffraction) and the range of grain sizes measured to obtain individual sample GSDs varies between studies (see “grain size” tab in Additional File 1). The equations used to calculate grain size statistical parameters such as mean diameter and sorting coefficient also differ between studies. Componentry data are inconsistently reported, as either wt% or proportion, and the criteria used for categorizing different components (e.g., juvenile, lithic) is not always clearly defined. Descriptions of sedimentary structures, bedforms, and lithofacies are often qualitative and are not measured, limiting numerical or statistical analysis. Quantitative measurements of bedforms also vary in format, with some authors documenting wavelength/amplitude and others length/height (Fig. 3c). These inconsistencies restrict the number of PDC deposits from different studies/volcanoes/eruptions that can be directly compared for some data categories.

Where studies report datasets such as GSDs and thickness only in figures, it was not always possible to accurately obtain these data for inclusion in the database; for example, if GSD histogram figures or thickness isopach maps were of insufficient resolution for digital extraction. Hence, published PDC deposit data for some volcanoes/eruptions is not always easily obtained for re-use by the wider scientific community. In constructing the database, we observed that few studies report uncertainties for quantitative data and grain size statistical parameters obtained from PDC deposits. Therefore, it is currently difficult to incorporate uncertainty on field/laboratory constrained parameters into numerical simulations of PDCs.

## Conclusions and future developments

The PDCD-DAT database (Brown et al. 2026) records both quantitative measurements from and qualitative descriptions of PDC deposits, representing 214 individual depositional units formed during 97 eruptions at 55 volcanoes distributed globally.

The database provides a valuable resource for improving numerical and analogue modelling of PDCs. Users can extract data from deposits with specific characteristics and/or from specific volcanoes and/or from eruptions of different magnitudes, to obtain well-constrained model input parameters and compare with model outputs for validation. Ultimately, the development of new and improved models has the potential to drive advances in understanding of the links between PDC dynamics and resulting deposits, as well as our ability to forecast and mitigate against the hazards posed by PDCs.

Some eruptions in PDCD-DAT have corresponding data on eruption source parameters in the Independent Volcanic Eruption Source Parameter Archive (IVESPA) database (Aubry et al. 2021), which are used as inputs for numerical models of explosive eruption columns. Therefore, data from PDCD-DAT could be combined with IVESPA datasets to facilitate numerical modelling of past explosive eruptions involving both tephra fallout and PDC forming phases.

Some datasets for individual volcanoes can be used to estimate hazard impact metrics of PDCs from previous eruptions, providing valuable information for hazard assessment purposes. The database also enables users to compare deposits from different volcanoes and/or eruptions using many different criteria, to identify common trends and individual volcanoes/events where PDCs produced deposits with distinctive properties. Further investigation of the factors controlling common trends in PDC deposit characteristics may yield new insights into fundamental aspects of PDC behaviour and the interpretation of their deposits.

The integration of PDCD-DAT with FlowDat provides a sustainable platform for the database. PDCD-DAT is hosted on a dedicated section of the developing FlowDat website (https://flowdat.org/pdcd-dat/). Currently, the PDCD-DAT data can be downloaded, and informational pages explain the contents of the database. We hope that the PDCD-DAT webpage will provide a user interface for searching and filtering the database in the future.

We hope that PDCD-DAT will be expanded in the future, through addition of other existing published datasets and datasets from new field studies. We strongly encourage authors of future PDC field studies, or of studies not yet included in the database, to consider submitting their datasets for incorporation into the database. We provide a “data import template” for authors to submit their datasets in Additional File 3, which can be e-mailed to the corresponding author, along with any queries. The template makes recommendations on how these data, and the methods of data collection, should be reported. The template is also available on the PDCD-DAT webpage. New entries to the database could be moderated by the corresponding author. Future submissions of new, high-quality datasets could increase both the quantity and quality of the data available in PDCD-DAT, adding to its value as a resource for the scientific community studying PDCs.

Finally, in compiling this database we make the observations that (1) there is no consistent approach to characterisation, measurement, or sampling of deposits in the field; (2) a proliferation of laboratory techniques has led to increased quantification of sample characteristics, but there is a lack of standard reporting of methodologies, including uncertainty reporting; (3) the highly variable nature of deposits is rarely captured in publications reporting deposit properties. There is a need for the community to develop a rigorous approach to field data collection, analysis, and reporting, which is currently being addressed in ongoing work by this team.

## Supplementary Information

Below is the link to the electronic supplementary material.

Supplementary Material 1: PDCD-DAT Flat File database – A spreadsheet containing the complete PDCD-DAT database.

Supplementary Material 2: PDC deposit properties definitions – A spreadsheet listing definitions for each PDC deposit property recorded in the database. Each property corresponds to a column in the database spreadsheet (Additional File 1).

Supplementary Material 3: Data import template – A blank spreadsheet with column headers corresponding to the PDC deposit properties recorded in the database. Researchers can download the spreadsheet and populate it with their field datasets, which can then be e-mailed to the corresponding author for inclusion in the database.

Supplementary Material 4: Map - A figure showing a world map of the volcanoes featured in PDCD-DAT.

Supplementary Material 5: Database content summary - A spreadsheet listing the number of data entries for each PDC deposit property recorded in the database.

Supplementary Material 6: Figure displaying the PDC deposit properties recorded under each category in the database.

## Data Availability

The datasets supporting the conclusions of this article are included within the article and its additional files. PDCD-DAT can be accessed through the FlowDat website at https://flowdat.org/pdcd-dat/. Additionally, the PDCD-DAT database has been deposited in the National Geoscience Data Centre (NGDC), the designated NERC (Natural Environment Research Council, part of UK Research and Innovation) data centre for the storage and management of geoscience data in the UK (Brown et al. 2026). Hosted by the British Geological Survey (BGS), the NGDC is certified as a trusted repository by CoreTrustSeal.

## Abbreviations

PDC: Pyroclastic density current
GSD: Grain size distribution
BAF: Block and Ash flow
H/L: Ratio of height descended (H) to PDC runout (L)
