# Supplementary figures and images for: PDCD-DAT – a global database of pyroclastic density current deposit field data
Source: J Appl Volcanol. 2026 May 11;15(1):9. doi: 10.1186/s13617-026-00167-6 (PMC13158253; doi:10.1186/s13617-026-00167-6)

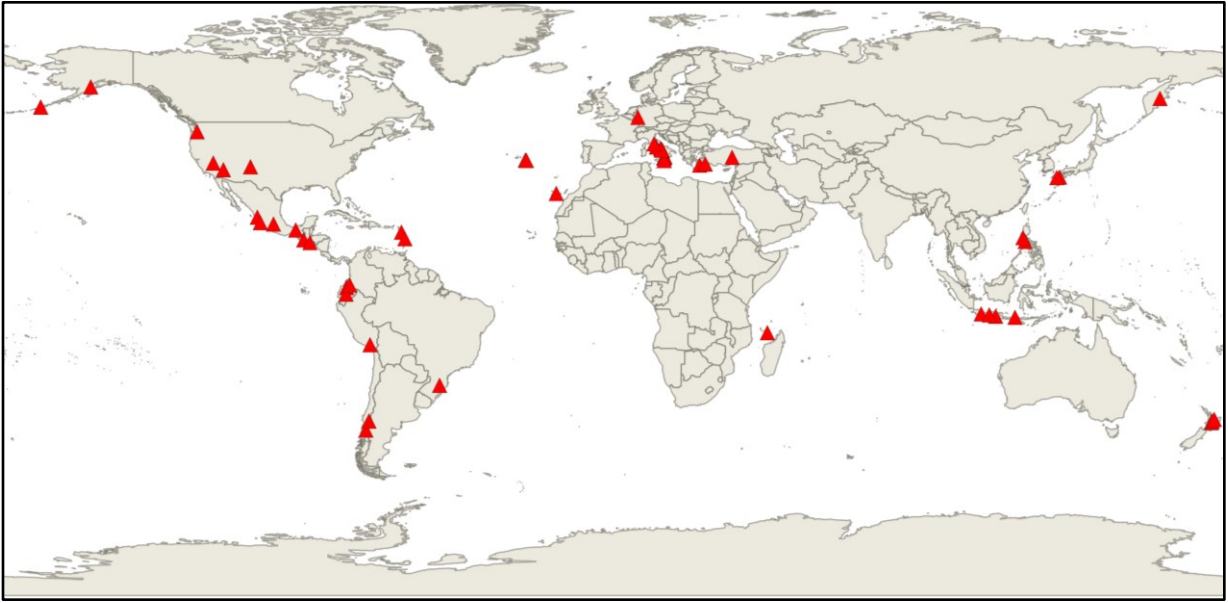

**Additional Figure 1:** Map showing global distribution of volcanoes featured in PDCCD-Dat.

Supplement: Supplementary file 4 — Supplementary Material 4: Map - A figure showing a world map of the volcanoes featured in PDCD-DAT. [file 13617_2026_167_MOESM4_ESM.pdf]
