# Supplementary material for: PDCD-DAT – a global database of pyroclastic density current deposit field data
Source: J Appl Volcanol. 2026 May 11;15(1):9. doi: 10.1186/s13617-026-00167-6 (PMC13158253; doi:10.1186/s13617-026-00167-6)
Supplement: Supplementary file 6 — Supplementary Material 6: Figure displaying the PDC deposit properties recorded under each category in the database. [file 13617_2026_167_MOESM6_ESM.pdf]

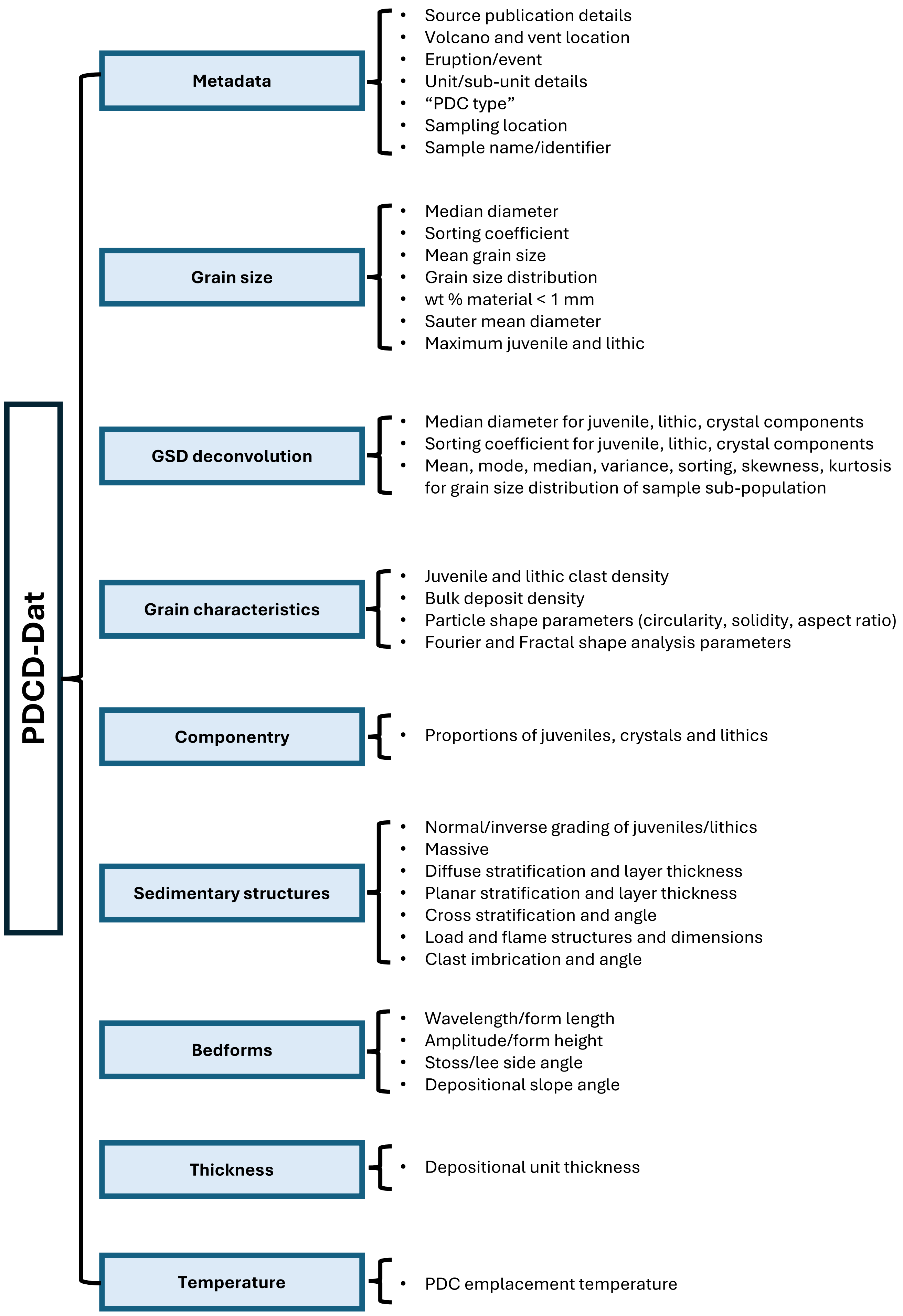

Additional Figure 2: Diagram summarising the PDC deposit properties recorded under each category in the database.
